# Renal Leiomyosarcoma, a Rare Presentation

**DOI:** 10.15586/jkcvhl.v9i1.216

**Published:** 2022-03-21

**Authors:** Toukilnan Djiwa, Kossi Akomola Sabi, Panakinao Simgban, Mayi Bombonne, Bagassam Mézéwè Sama, Mazamaesso Tchaou, Tchin Darré

**Affiliations:** 1 Department of Pathological Anatomy, Teaching Hospital of Lomé, Togo;; 2 Department of Nephrology, Teaching Hospital of Lomé, Togo;; 3 Department of Imaging, Teaching Hospital of Lomé and Kara, Togo

**Keywords:** kidney, immunohistochemistry, leiomyosarcoma, Nephrology

## Abstract

Renal sarcomas are very rare malignant tumours with a very poor prognosis. Renal leiomyosarcoma, a malignant tumour of smooth muscle origin, is the most common histological type. The article reports a case of leiomyosarcoma of renal location, with a review of the literature. A 38-year-old female patient, with no previous pathological history, consulted the nephrology department of the Teaching Hospital of Lomé for abdominal pain that had been present for 4 years. Histology showed a tumour proliferation of fasciculated architecture, made of spindle cells arranged in long bundles, with cytonuclear atypia and cytoarchitectural abnormalities. Immunohistochemical examination showed positive staining for smooth muscle actin, h-caldesmone, desmin and CD34 and negative for pancytokeratin (AE1/AE3), myogenin and PS100. Renal leiomyosarcoma is an exceptional malignancy. It remains the most common renal sarcoma, the differential diagnosis of which is based on immunohistochemical findings.

## Introduction

Renal sarcomas represent 1–3% of malignant tumours of the kidney, frequently occurring in the fifth or sixth decade of life (1). Leiomyosarcoma remains the most common histological type accounting for 50–60% of renal sarcomas (2). It is a malignant tumour of smooth muscle origin that usually occurs in soft tissue and the uterus. However, a small percentage may originate from smooth muscle or vascular walls, most of which are venous in origin (3). Renal leiomyosarcomas can arise from the smooth muscle fibres of the renal pelvis and the renal capsule or the renal vessels, the latter being more common (4).

We report a case of renal leiomyosarcoma, describe the clinical presentation and diagnostic features and discuss the prognostic and therapeutic features of primary renal leiomyosarcomas.

## Case Report

A 38-year-old woman with no previous pathological history consulted the nephrology department of the Teaching Hospital of Lomé for abdominal pain that had been present for 4 years and had been considered as renal colic.

**Figure 1:**
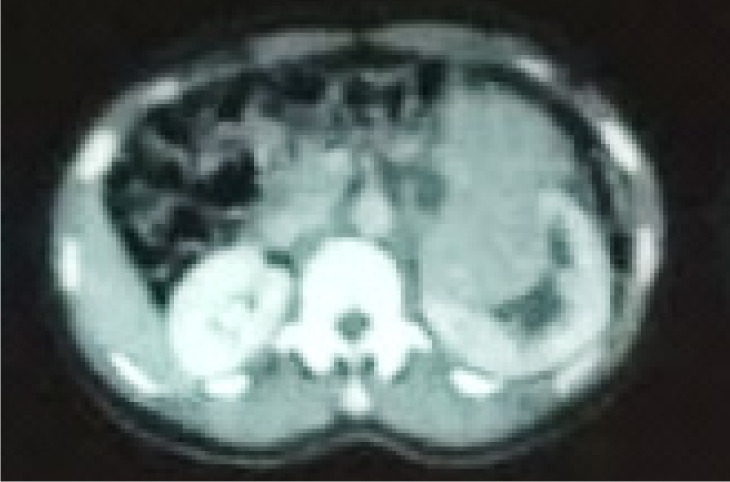
Abdominal CT scan showing a suspicious tissue mass in the left kidney.

**Figure 2:**
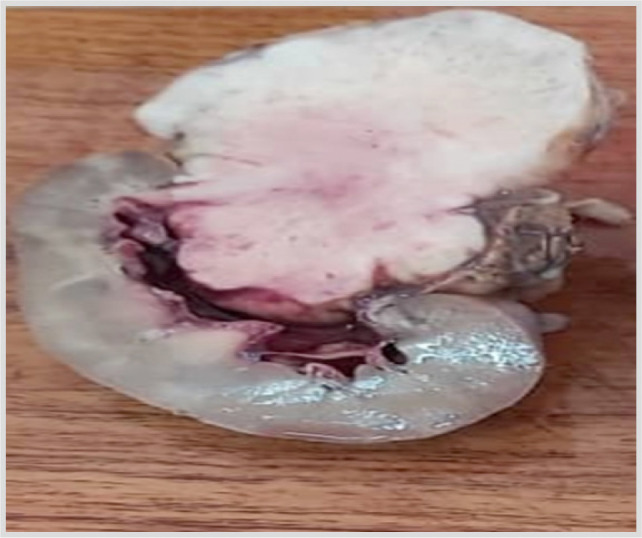
Macroscopic appearance of the sample after opening.

The clinical examination revealed an axillary temperature of 37.4°C, a body weight of 56 kg for a height of 1.67 m. Blood pressure was 120/70 mmHg. The patient was in good general condition with well-stained conjunctiva. A painful mass was palpated on the left flank. The lymph nodes were free. The biological workup included a normal blood count, uremia, creatinine and blood glucose, all of which were returned normal. A computed tomography (CT) scan revealed a suspicious tissue mass measuring 9 × 3 cm, with homolateral hydrocalice and without any secondary thoraco-abdominal lesion (Figure 1). The paraclinical workup was completed by a uroscanner which revealed a spontaneously isodense process in the left kidney, enhanced after injection of contrast medium, without extension to the renal vein, resulting in stage III left ureterohydronephrosis with excretory disorder. The right kidney and intra-abdominal solid organs were without abnormalities. There was a large polymyomatous uterus with a normal bladder. An extended nephrectomy was performed and the specimen was sent to the pathology laboratory. Macroscopically, it was a nephrectomy specimen, without a ureter, weighing 640 g, measuring 15 × 12 × 5 cm, with a bumpy surface and an elastic consistency. On section, there was a well-limited whitish tumour lesion measuring 12 × 12 × 4 cm with haemorrhagic changes and a peripheral healthy portion of 3 cm without peri-renal fat infiltration (Figure 2).

**Figure 3:**
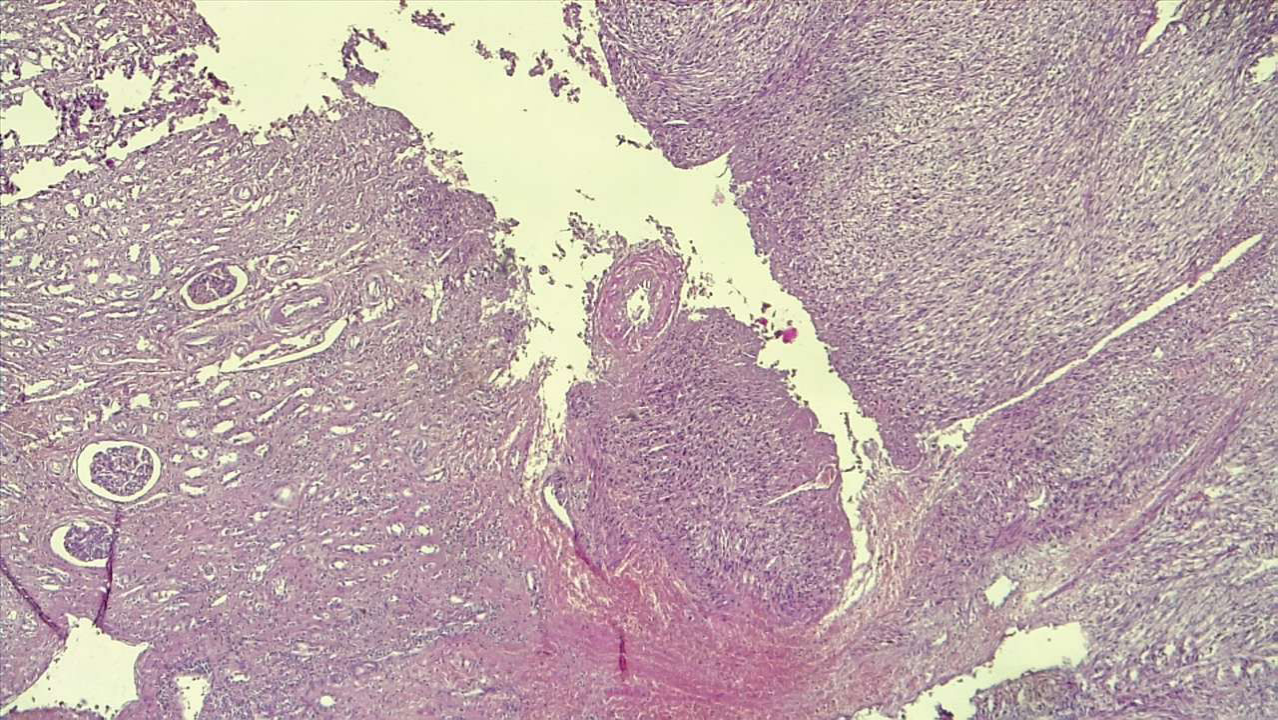
(HE × 40). Normal kidney (left) and fasciculated tumour proliferation (right).

**Figure 4:**
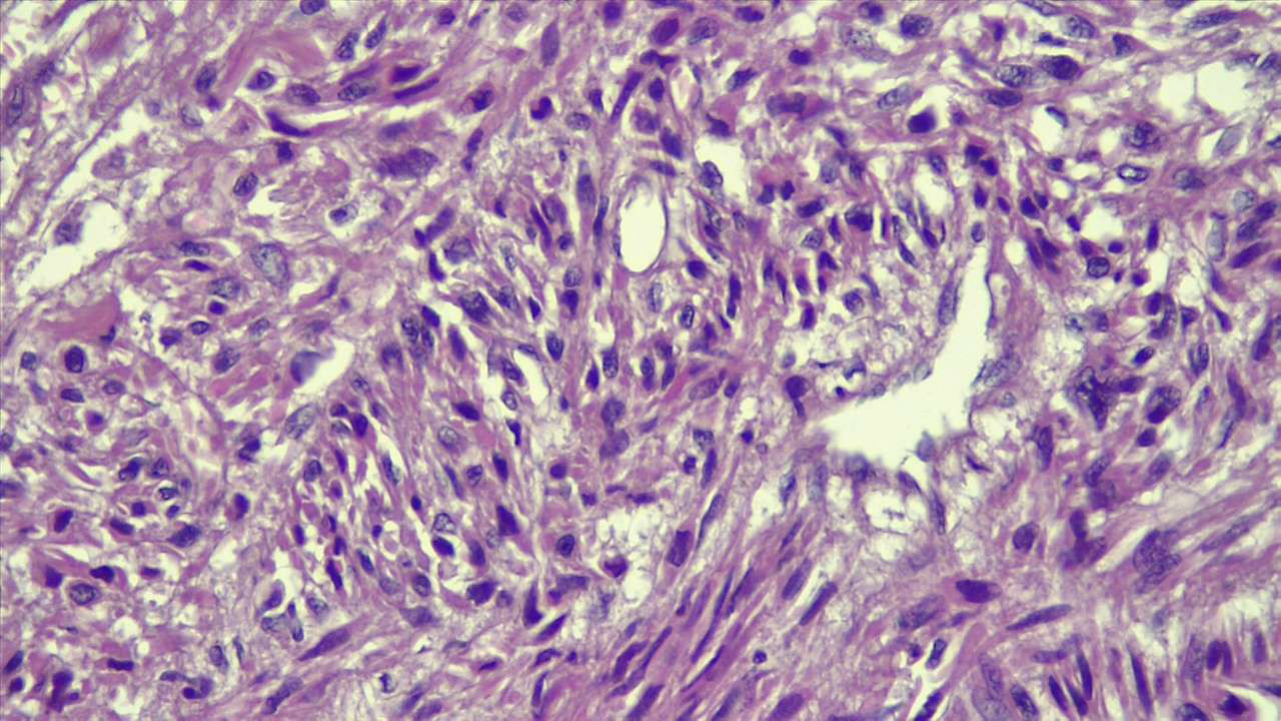
(HE × 400). Spindle cell proliferation with clear anisokaryosis.

The resected specimen was treated with 10% formaldehyde, followed by conventional dehydration, paraffin embedding, sectioning and hematoxylin and eosin (HE) staining. Histology showed a tumour proliferation with a fasciculated architecture, made of spindle cells arranged in long bundles, showing cytonuclear atypia and cytoarchitectural abnormalities. The cells had eosinophilic cytoplasm, an elongated nucleus, marked anisokaryosis and mitotic rate estimated at 9 mitoses/10 HPF in hotspot areas. No carcinomatous contingent or necrosis was found (Figures 3 and 4). Due to this, we suggested as diagnoses, a renal fibrosarcoma, an inclined renal carcinoma with a sarcomatoid component estimated at 100%, a renal synovialosarcoma and a renal leiomyosarcoma in the last instance. Immunohistochemical examination showed intense and diffuse labelling of actin, h-caldesmone and desmin and intense and focal labelling of CD34. There was an absence of immunostaining for pan-cytokeratin AE1/AE3, myogenin and S100 protein. In view of this immunomorphological profile, we made the diagnosis of FNCLCC grade 2 renal leiomyosarcoma (3 + 1 + 0).

The patient has no evidence of recurrence after a 16-month follow-up.

## Discussion

### Epidemiology

Renal sarcomas represent 1–3% of adult renal malignancies (1). Leiomyosarcomas constitute 50–60% of renal sarcomas (2, 5). These tumours usually arise from the renal capsule, the smooth muscle tissue of the renal pelvis and the intra-renal vessels (4, 5). They occur predominantly in the fifth or sixth decade of life, with an incidence that increases with age (1, 6, 7). Renal leiomyosarcoma is more common in women, most often occurring in the right kidney (6).

### Clinic and imaging

Renal leiomyosarcomas usually have an insidious clinical presentation with late-stage symptoms of abdominal pain, vomiting, weight loss and a palpable mass (8). Compression of neighbouring organs causes back pain and haematuria (6). Neither ultrasound, CT scan, nor MRI can differentiate leiomyosarcomas from renal cell carcinomas (9).

### Histopathology

On microscopic examination, leiomyosarcomas have the characteristics of a smooth muscle tumour with a fasciculated architecture, made up of spindle-shaped cells arranged in long tangled bundles. The cells have sharp-ended, non-tapered nuclei with eosinophilic cytoplasm. Indicators of malignancy are necrosis, nuclear pleomorphism with rare mitotic figures (1). It is extremely difficult to differentiate a renal leiomyosarcoma from a renal sarcomatoid carcinoma. Both tumours have similar clinical, radiographic and pathological features (4). Only the absence of an epithelial contingent on morphological examination and the absence of cytokeratin expression on immunohistochemical examination can formally rule out a sarcomatoid carcinoma of the kidney (10, 11). Primary monophasic synovial sarcoma of the kidney also shows monophasic spindle cells. The spindle cells are plump with irregular cell borders. They tend to grow in sheets and usually have trapped renal tubules in the form of cysts. However, these tumours show positivity for Bcl-2 (1).

Immunohistochemistry reveals positive immunostaining for smooth muscle actin (SMA), h-caldesmone, desmin and vimentin. They are negative for epithelial markers (4).

### Treatment and prognosis

Renal leiomyosarcomas are generally biologically aggressive with a poor prognosis. Radical nephrectomy is the treatment of choice (12). The major prognostic factor is total surgical resection (12). Lewis et al. reported that the presence of unresectable disease and incomplete surgical resection were the most important predictors of disease-specific death (13).

Life expectancy is lower for renal sarcomas than for other urinary tract sarcomas. The 5-year survival is 82% for patients with retroperitoneal sarcoma, 73% for bladder sarcoma, 44% for prostate sarcoma and 39% for patients with renal sarcoma (14). According to Geonseok et al., 5-year survival rate is 51.4% for all urogenital sarcomas (14).

Our patient underwent radical and extended nephrectomy. She is free of recurrence after a 16-month follow-up.

## Conclusion

Renal leiomyosarcomas are rare malignant mesenchymal tumours with a poor prognosis. They usually arise from the renal capsule, the smooth muscle tissue of the renal pelvis and the intra-renal vessels. They can only be differentiated from sarcomatoid renal cell carcinoma by immunohistochemical examination. Radical nephrectomy is the standard treatment and total surgical resection is the major prognostic factor.

## Statements

### Ethical Approval and Consent to Participate

The study received approval from the head of the laboratory to be conducted. The manuscript was not submitted to more than one journal for simultaneous review and has not been previously published. Single study, not divided into multiple parts and no data were generated.

### Consent for Publication

The Department of Pathology of the Teaching Hospital of Lomé has authorized the publication of this manuscript (Ref No. 18/2021/LAP/CHUSO).

## Data Availability

All data supporting the conclusions of this article are included in the manuscript and its supporting documents.
